# Clinical Characteristics and Associated Comorbidities in Children and Adolescents With Type 1 Diabetes Mellitus: A Retrospective Study

**DOI:** 10.7759/cureus.109304

**Published:** 2026-05-20

**Authors:** Salman Almansour

**Affiliations:** 1 Pediatric Endocrinology, Qassim University, Buraydah, SAU

**Keywords:** comorbidities, glycemic control, pediatrics, saudi arabia, type 1 diabetes mellitus

## Abstract

Objective: Type 1 diabetes mellitus (T1DM) in pediatric and adolescent populations is correlated with significant metabolic instability and an elevated risk of autoimmune and metabolic comorbidities, which can negatively impact long-term health outcomes. Notwithstanding the increasing prevalence of pediatric T1DM in Saudi Arabia, there is a paucity of region-specific evidence delineating the clinical profiles and associated comorbid conditions within this demographic. This study aimed to evaluate the clinical features and comorbidities among children and adolescents with T1DM.

Methods: This retrospective analysis was conducted from March 2020 to February 2023 at the Pediatrics Department of King Saud Hospital in Unaizah, Qassim region, Saudi Arabia. The study included Saudi children aged two to 17 years diagnosed with T1DM. Sociodemographic, anthropometric, and laboratory data were extracted. Data were analyzed using SPSS v22 (IBM Corp., Armonk, NY, USA). Descriptive statistics were used, along with paired-samples t-tests to assess changes in glycated hemoglobin (HbA1c) and chi-square tests to examine associations between variables.

Results: This study included 407 children and adolescents with T1DM. There were 211 males (51.8%) and 196 females (48.2%). The mean age was 12.27±4.54 years, with a mean age at diagnosis of 7.92±3.94 years. Poor glycemic control at diagnosis (HbA1c ≥ 9%) was observed in 288/370 patients (77.8%), which decreased to 222/375 patients (59.2%) at final follow-up, with a mean HbA1c reduction of 0.83%. A family history of T1DM was noted in 50/131 patients (38.2%), and type 2 diabetes in 83/121 patients (68.6%). The prevalence of poor glycemic control (HbA1c ≥9%) differed significantly across age groups, with higher rates observed among adolescents aged 10-15 years (67.6%) and ≥15 years (64.9%) compared with children aged <10 years (36.3%) (p<0.001).

Conclusion: Children and adolescents with T1DM were found to have poor glycemic control and a high rate of autoimmune and metabolic comorbidities, particularly thyroid dysfunction, dyslipidemia, and vitamin D deficiency. These findings highlight the need for systematic screening and comprehensive clinical monitoring in pediatric populations with T1DM.

## Introduction

Type 1 diabetes mellitus (T1DM) is an autoimmune disorder characterized by the destruction of pancreatic beta cells, leading to lifelong insulin deficiency and necessitating exogenous insulin replacement [1]. It is a rapidly progressing condition in children and adolescents and is marked by clinical symptoms linked to hyperglycemia and metabolic imbalance [2]. It typically begins during periods of significant growth, specifically between the ages of 4-6 or 10-14. This presents challenges in treatment, as insulin requirements fluctuate in response to puberty and physical activity [1,2]. Genetic predisposition plays a significant role, with human leukocyte antigen (HLA)-DR3 and HLA-DR4 alleles frequently linked to the disease; however, environmental factors, including viral infections, may trigger rapid beta-cell destruction [3].

In comparison with type 2 diabetes, T1DM among youth has not been associated with obesity as a primary cause. Recent trends indicate an increasing overlap with metabolic factors, which may complicate glycemic regulation [4]. Globally, approximately 9.2 million people have T1DM, including 1.8 million under age 20, with childhood incidence rates increasing by 3% per year in many areas [5]. In Saudi Arabia, an estimated 35000 children and adolescents are affected, with an incidence of 33.5 per 100000, which is one of the highest in the world [6]. Understanding the clinical presentation of T1DM in children is essential for early diagnosis and prevention of acute complications.

Children with T1DM typically present with the classic symptoms of polyuria, polydipsia, weight loss despite normal or increased appetite, and lethargy, primarily due to hyperglycemia-induced osmotic diuresis and cellular energy deficiency [7]. About 38.5% of new pediatric cases present with diabetic ketoacidosis (DKA), a critical condition with acidosis, volume depletion, and ketonemia due to unchecked lipolysis [8]. Nocturia and enuresis may occur in previously toilet-trained children, a sign of advanced hyperglycemia. Fatigue, irritability, and blurred vision are common and represent the systemic effects of increased glucose levels. Adolescents may present with more subtle signs; however, if undiagnosed, they can become severely dehydrated and develop acidosis. Laboratory findings typically reveal hyperglycemia above 200 mg/dL, reduced C-peptide levels, and autoantibodies such as glutamic acid decarboxylase 65 (GAD65), islet antigen-2 (IA-2), and islet cell antibodies [9]. In specific populations, such as individuals with Down syndrome, the development of T1DM may occur earlier (approximately 4.9 years), with lower insulin requirements and similar rates of ketoacidosis [10].

Evidence indicates that one in three children with T1DM is diagnosed with at least one comorbidity during their lifetime [11]. These comorbid autoimmune disorders make diabetes management in young individuals more complex. Between 17% and 30% of children with T1DM also have autoimmune thyroiditis, which often presents as subclinical thyroid dysfunction and can affect growth and metabolic balance if left untreated [12]. Celiac disease co-occurs in 9.8% of cases, leading to malnutrition, glycemic instability, and delayed puberty without gluten-free intervention [13].

Other autoimmune comorbidities include gastritis (with cumulative risks increasing with the duration of the disease) and Addison's disease [12]. Children also experience early cardiovascular risk factors, including dyslipidemia and hypertension, with multiple risks converging to favor the development of endothelial dysfunction and early atherosclerosis [11]. Microvascular complications such as neuropathy, retinopathy, and nephropathy can arise even during adolescence, depending on cumulative hyperglycemia and disease duration [14]. In addition to medical complications, psychosocial and familial factors significantly contribute to the disease burden associated with pediatric T1DM.

Psychiatric comorbidities are also associated with T1DM in youth, with depression and anxiety placing affected individuals at risk of poor metabolic control. Elevated glycated hemoglobin (HbA1c) levels and hospitalization following ketoacidosis have been linked to disordered eating behaviors [2]. Neurodevelopmental conditions affect others, influencing academic performance and control. In Saudi Arabia, T1DM within families is observed in 19.5% of cases and is associated with earlier onset and a higher proportion of insulin autoantibody-positive cases [15]. These comorbidities demand holistic treatment, as they lead to treatment burden and reduced quality of life. Early detection of related illnesses is also important to prevent ketoacidosis [15]. Despite the acknowledged clinical and comorbid burden associated with pediatric T1DM, region-specific data in Saudi Arabia remain scarce.

In Saudi Arabia, pediatric T1DM data show regional variation in incidence and limited data on comorbidity patterns. There is insufficient evidence regarding either epidemiological prevalence or individual outcomes, including DKA or autoimmune comorbidities. This research aims to evaluate clinical characteristics and comorbidities among Saudi children and adolescents with T1DM to identify local trends that can inform management plans.

## Materials and methods

This retrospective observational study was conducted in the Pediatrics Department of King Saud Hospital, Unaizah, Qassim Region, Saudi Arabia. Medical records of children and adolescents diagnosed with T1DM between March 2020 and February 2023 were reviewed. The study population comprised Saudi children aged 2-17 years with a confirmed diagnosis of T1DM based on clinical presentation and biochemical criteria documented in hospital records. Patients with incomplete core clinical data or secondary causes of diabetes were excluded.

The sample size was estimated using a single proportion formula, assuming a 95% confidence level (Z=1.96), a margin of error of 5%, and a prevalence of 50% to ensure maximum sample size. The minimum required sample size was calculated to be 384 participants. The final sample of 407 patients exceeded this requirement.

Data were collected utilizing a structured data abstraction form specifically designed for this study. Sociodemographic, clinical, anthropometric, and laboratory data were retrospectively extracted from electronic and paper medical records using a structured data abstraction form. HbA1c values at diagnosis were obtained from the initial presentation, while follow-up HbA1c reflected the most recent clinic measurement documented in the medical record. The collected variables encompassed age, sex, age at diagnosis, estimated disease duration, family history of diabetes mellitus, and parental consanguinity. Anthropometric measurements (height and weight) were documented at diagnosis and at the most recent clinic visit, employing standardized clinical procedures during routine outpatient appointments. Body mass index (BMI) was calculated as weight in kilograms divided by height in meters squared (kg/m²). Paired anthropometric analyses were conducted for patients with available measurements at both time points. Data extraction was conducted by trained members of the research team under the supervision of the principal investigator. To ensure accuracy and consistency, a subset of records was independently reviewed, and any discrepancies were resolved through discussion and verification with the original medical records.

The comorbidities assessed in this study included dyslipidemia, thyroid dysfunction, celiac disease, vitamin D deficiency, diabetic retinopathy, and microalbuminuria. Data were analyzed using the Statistical Package for the Social Sciences (SPSS), version 22 (IBM Corp., Armonk, NY, USA). Continuous variables were expressed as mean±standard deviation or median. Categorical variables were presented as frequencies and percentages. Analyses were performed using available-case analysis due to incomplete data inherent to retrospective medical record review; therefore, denominators vary across variables. Longitudinal changes in glycemic control were evaluated through paired-samples t-tests. Additionally, the associations between categorical variables and glycemic control status at follow-up were analyzed using Pearson's chi-square test. Poor glycemic control was defined as HbA1c ≥9%, consistent with American Diabetes Association (ADA) recommendations for pediatric diabetes management [16].

The research was conducted in accordance with local legislation and institutional requirements. Ethical approval was received from the Regional Research Ethics Committee of Qassim Province, Saudi Arabia, under Reference No. 607456181.

## Results

A total of 407 children and adolescents with T1DM were included in the analysis. Among these participants, 211 (51.8%) were male, and 196 (48.2%) were female. The mean current age was 12.27±4.54 years (median: 13; interquartile range (IQR): 9-16).

Age at diagnosis was available for 382 patients, with an average of 7.92±3.94 years (median: 8; IQR: 5-11). Within this cohort, 89 (23.3%) were diagnosed before the age of five years, 161 (42.1%) between the ages of five and nine years, 110 (28.8%) between 10 and 14 years, and 22 (5.8%) at or after the age of 15 years. The estimated mean follow-up duration was 4.49±3.52 years (median: 4; IQR: 1-6). Consanguinity data were available for 100 patients, and 49 (49.0%) reported parental consanguinity (Table 1).

**Table 1:** Baseline demographic and clinical characteristics of the study population (n=407) ^*^Data on age at diagnosis and follow-up duration were available for 382 patients due to incomplete retrospective records. IQR: interquartile range

Variable	Value
Total patients, n	407
Sex, n (%)	
Male	211 (51.8)
Female	196 (48.2)
Current age (years)	
Mean±SD	12.27±4.54
Median (IQR)	13 (9-16)
Age at diagnosis (years)^*^ (n=382)	
Mean±SD	7.92±3.94
Median (IQR)	8 (5-11)
Age at diagnosis categories (n=382), n (%)	
<5 years	89 (23.3)
5-9 years	161 (42.1)
10-14 years	110 (28.8)
≥15 years	22 (5.8)
Estimated follow-up duration (years)^*^	
Mean±SD	4.49±3.52
Median (IQR)	4 (1-6)
Parental consanguinity (recorded n=100) n (%)	
Yes	49 (49.0)
No	51 (51.0)

A family history of T1DM was documented for 131 patients, with 50 (38.2%) indicating a positive family history. Among those with available counts (n=44), the mean number of affected relatives was 1.75±0.94 (median: 1; range: 1-4). Family history of type 2 diabetes mellitus (T2DM) was available for 121 patients, of whom 83 (68.6%) reported a positive history. The mean number of affected relatives among those with documented counts (n=76) was 2.01±0.99 (median: 2; range: 1-5) (Table 2).

**Table 2:** Family history of diabetes mellitus T2DM: type 2 diabetes mellitus; T1DM: type 1 diabetes mellitus

Variable	Value
Family history of T1DM (recorded, n=131) n (%)	
Yes	50 (38.2)
No	81 (61.8)
Number of affected relatives (T1DM, n=44)	
Mean±SD	1.75±0.94
Median (range)	1 (1-4)
Family history of T2DM (recorded, n=121) n (%)	
Yes	83 (68.6)
No	38 (31.4)
Number of affected relatives (T2DM, n=76)	
Mean±SD	2.01±0.99
Median (range)	2 (1-5)

Over a mean follow-up period of 3.82 years, mean HbA1c decreased from 10.45±1.99% at diagnosis to 9.62±1.94% at last follow-up, corresponding to a mean reduction of 0.83%. This decrease was statistically significant (t(338)=6.28, p<0.001; 95% CI: 0.57-1.09) (Table 3).

**Table 3:** Glycemic control in children and adolescents with T1DM Independent sample t-test HbA1c: glycated hemoglobin; T1DM: type 1 diabetes mellitus

HbA1c	n	Mean±SD	Mean difference (%)	t (df)	95% CI of difference	P-value
At diagnosis	339	10.45±1.99	0.83	6.28 (338)	0.57-1.09	<0.001
At last follow-up	339	9.62±1.94				

Glycemic control differed significantly across age groups, with HbA1c ≤9% observed in 63.7% of children aged <10 years compared with 32.4% in those aged 10-15 years and 35.1% in those aged ≥15 years; correspondingly, HbA1c >9% was present in 36.3%, 67.6%, and 64.9% of patients, respectively (χ²=27.71, p<0.001). The difference between sexes was not statistically significant (χ²=0.83, p=0.361), suggesting that sex was not associated with glycemic control at follow-up (Table 4).

**Table 4:** Association between current age group and glycemic control at follow-up Association between age groups and glycemic control assessed using Pearson’s chi-square test of independence. HbA1c: glycated hemoglobin

Variable		HbA1c ≤9%, n (%)	HbA1c >9%, n (%)	Total (n)	Pearson chi-square, p-value
Age group	<10 years	65 (63.7)	37 (36.3)	102	χ²=27.71, p<0.001
	10-15 years	45 (32.4)	94 (67.6)	139	
	≥15 years	47 (35.1)	87 (64.9)	134	
	Total	157	218	375	
Gender	Male	86 (44.1)	109 (55.9)	195	χ²=0.83, p=0.361
	Female	71 (39.4)	109 (60.6)	180	
	Total	157	218	375	

The mean total cholesterol level was 4.65±1.08 mmol/L, with elevated levels detected in 57 out of 220 participants (25.9%). The mean low-density lipoprotein (LDL) cholesterol was 2.87±0.89 mmol/L, with 47 of 198 participants (23.7%) having high LDL cholesterol. The mean high-density lipoprotein (HDL) cholesterol was 1.52±0.41 mmol/L, with low levels identified in 17 out of 193 participants (8.8%). The mean triglyceride concentration was 1.25±0.93 mmol/L (median: 0.98; IQR: 0.70-1.50), with elevated levels present in 57 out of 213 participants (26.8%). Thyroid function assessment revealed a mean thyroid-stimulating hormone (TSH) level of 5.03±15.13 mIU/L, with TSH levels greater than 4.0 mIU/L in 102 out of 391 participants (26.1%). Additionally, the mean free thyroxine (FT4) concentration was 16.18±3.08 pmol/L (median 16.19; IQR 14.25-18.14), with reduced FT4 levels noted in 26 out of 388 participants (6.7%) (Table 5).

**Table 5:** Lipid profile and thyroid function parameters ^†^Triglyceride abnormality defined using pediatric age-adjusted thresholds. Values are presented as mean±SD, median (IQR), or n (%). Laboratory reference ranges were age-adjusted where applicable. LDL: low-density lipoprotein; HDL: high-density lipoprotein; TSH: thyroid-stimulating hormone; FT4: free thyroxine; IQR: interquartile range; HbA1c: glycated hemoglobin

Parameter	n	Mean±SD	Median (IQR)	Abnormal, n (%)
Lipid profile (mmol/L)				
Total cholesterol	220	4.65±1.08	4.45 (3.90-5.24)	High (≥5.2): 57 (25.9)
LDL cholesterol	198	2.87±0.89	2.75 (2.26-3.31)	High (≥3.4): 47 (23.7)
HDL cholesterol	193	1.52±0.41	1.47 (1.25-1.73)	Low (<1.0): 17 (8.8)
Triglycerides	213	1.25±0.93	0.98 (0.70-1.50)	High^†^: 57 (26.8)
Thyroid function				
TSH (mIU/L)	391	5.03±15.13	2.54 (1.60-4.17)	>4.0: 102 (26.1)
FT4 (pmol/L)	388	16.18±3.08	16.19 (14.25-18.14)	<12: 26 (6.7)

Retinopathy status was documented in 55 patients, with evidence of diabetic retinopathy in 5/55 (9.1%). Urine albumin-to-creatinine ratio was available for 160 patients (median: 0.15; IQR: 0.10-0.24), with elevated values observed in 3/160 (1.9%). Celiac disease screening was documented in 95 patients, of whom 13/95 (13.7%) tested positive. Vitamin D levels were available for 339 patients, with a mean concentration of 49.77±23.88 nmol/L (median: 46.11; IQR: 32.84-60.25). Vitamin D deficiency (<50 nmol/L) was observed in 192/339 (56.6%), insufficiency in 104/339 (30.7%), and sufficiency in only 43/339 (12.7%) (Table 6).

**Table 6:** Diabetes-related complications and associated comorbidities among children and adolescents with T1DM Screening performed according to the clinician's discretion T1DM: type 1 diabetes mellitus; ACR: albumin-to-creatinine ratio; IQR: interquartile range

Parameter	Available (n)	Measurement/summary	Abnormal n (%)
Diabetic retinopathy	55	-	5 (9.1)
Elevated urine ACR	160	Median 0.15 (IQR: 0.10-0.24)	3 (1.9)
Celiac disease	95	-	13 (13.7)
Serum vitamin D (nmol/L)	339	49.77±23.88; median 46.11 (32.84-60.25)	-
Vitamin D deficiency	339	-	192 (56.6)
Vitamin D insufficiency	339	-	104 (30.7)
Vitamin D sufficiency	339	-	43 (12.7)

## Discussion

The study assessed the clinical features and comorbidities of Saudi children and adolescents with T1DM in a Saudi tertiary-care setting, yielding several significant findings. Glycemic control at the time of diagnosis was generally suboptimal, characterized by markedly elevated HbA1c levels; however, a modest yet statistically significant improvement was observed during follow-up. Notably, glycemic control varied significantly across age groups, with adolescents demonstrating considerably higher rates of poor metabolic control than their younger counterparts. In contrast, no significant differences were observed between sexes. Furthermore, a substantial proportion of patients exhibited familial aggregation of diabetes and a high prevalence of metabolic and autoimmune comorbidities, including thyroid dysfunction, dyslipidemia, and vitamin D deficiency. Collectively, these findings underscore the persistent challenges in achieving optimal metabolic control during adolescence and highlight the necessity for systematic screening and age-targeted management strategies within pediatric populations affected by T1DM [1].

There was an almost equal sex distribution in our cohort (51.8% male, 48.2% female), consistent with a recent study by Abutaleb et al., which also reported a similar sex distribution (56.6% females) but no sex difference in familial cases [15]. The same study reported higher consanguinity (47.9%) in familial T1DM and associated it with earlier onset and more autoimmune comorbidities, findings that corroborate our finding of consanguinity in 49% of cases [15]. In contrast, a study conducted by Albishi et al. found no overall direct link between parental consanguinity and T1DM onset; however, first-cousin marriages slightly increased risk, contrasting with our higher rate [17].

In the current study, age is observed as a significant factor associated with glycemic control. Patents above 10 reported higher rates of poor metabolic control compared to their younger counterparts. In accordance with the findings of the current study, previous studies also reported similar age-related decline in glycemic outcomes, wherein factors such as pubertal insulin resistance, increasing treatment autonomy, and behavioral challenges contribute to the deterioration of HbA1c levels, despite advancements in diabetes management [18,19]

The present study identified 131 patients with a documented family history of T1DM, of whom 38.2% had a positive family history and had a mean of 1.75 affected relatives. Such prevalence may be compared with the Abutaleb et al. study, which identified the highest prevalence of 19.5% for family history of T1DM and 11.3% for siblings in the region, perhaps due to genetic clustering or the effect of consanguinity [15]. Nevertheless, AlMutair et al. reported a reduced prevalence of 15.9% in siblings [20]. These differences across studies may reflect variations in definitions of familial aggregation and in the reporting of affected relatives.

Among 121 patients with available data, 68.6% had a positive family history of type 2 diabetes, with a mean of 2.01 affected relatives. This is similar to a Swedish population-based study in which children with T1DM were 1.88 times more likely to have a parent with T2DM, indicating that common genetic or environmental pathways exist between diabetes types [21]. Nonetheless, it is compared to lower rates in the research by Parkkola et al., where 32%-52% of pediatric T1DM patients had extended family T2DM, perhaps because our study emphasized closer relatives and Saudi-specific lifestyle changes, such as increased obesity [22].

At diagnosis, the mean HbA1c of our 370 patients was 10.46%, with 77.8% having HbA1c ≥9%. This is similar to the 10.45% mean reported in the Saudi Pediatric and Youth Diabetes Registry (SPYDR), which included more than 2300 pediatric cases and indicated overall high initial values in Saudi centers [23]. In contrast, Alsaheel et al. reported a mean of 10.6%, whereas international data typically report slightly lower diagnostic levels (around 9%-11%) in well-resourced registries [24]. Females showed slightly higher HbA1c levels than males (9.77% vs. 9.44%), and a similar trend of significantly higher values in girls (10.88% vs. 10.3%) was observed by Alsaheel et al. [24].

In addition, lipid profiles showed mild dyslipidemia in our study. Catamo et al. found 32% dyslipidemia in children under 11, which declined to 18.5% in older children, consistent with our general abnormalities but not our age stratification [25]. Nonetheless, newer guidelines suggest screening after diagnosis with LDL >100 mg/dL and statin therapy when LDL is >130 mg/dL, findings supported by our data in a quarter of cases [2]. This shows a positive initial presentation, but there is an opportunity to enhance long-term management and lipid control to reduce early cardiovascular risk. These findings indicate early emergence of cardiovascular risk factors, supporting recommendations for routine lipid monitoring in pediatric T1DM.

In our cohort, thyroid function tests indicated elevated TSH levels in 26.1% of 391 patients, suggesting possible subclinical hypothyroidism, with 6.4% having elevated TSH, indicating overt hypothyroidism. FT4 disorders were less frequent: 6.7% below 12 pmol/L and 2.3% above 22 pmol/L. This is consistent with the report by Abutaleb et al., who found hypothyroidism in 6.7% of 987 T1DM children, but our more pronounced TSH elevation may reflect increased detection through systematic screening practices [15].

In 55 reported cases, retinopathy occurred in 9.1%, compared with 13.76% in a meta-analysis by Ezzatvar et al. in T1DM youth [14]. Renal involvement was 1.9%, compared with the 13.70% rate of nephropathy reported in the same meta-analysis [14]. In 95 cases, celiac positivity was 13.7%, compared to 8.3% in a Polish study, and consistent with Saudi findings of 9.2%, suggesting that shared HLA-related risks may increase celiac disease prevalence in Middle Eastern T1DM populations [15,26]. These findings support routine screening strategies recommended in pediatric T1DM guidelines.

The limitations of this study include its single-center design, which may not be representative of the broader Saudi population. The level of missing data indicates that comorbidities, including retinopathy and celiac disease, are not well-reported. The retrospective nature also limits causal inferences and necessitates prospective, multicenter studies.

## Conclusions

This study identified persistently suboptimal glycemic control among children and adolescents with T1DM, despite modest improvements observed during follow-up. Glycemic control varied significantly by age, with adolescents demonstrating higher rates of poor metabolic control than younger children; however, no differences were observed between sexes. The prevalence of autoimmune and metabolic comorbidities, particularly thyroid dysfunction, dyslipidemia, celiac disease, and vitamin D deficiency, was notable. These findings underscore the necessity for routine screening of comorbidities and the implementation of age-specific management strategies aimed at enhancing metabolic outcomes in pediatric populations with T1DM. Further prospective multicenter studies are warranted to validate these findings.
